# Irinotecan Hydrochloride Administration Considering Dosing-Time Attenuates Delayed Diarrhea in Rats

**DOI:** 10.3390/pharmaceutics18060645

**Published:** 2026-05-24

**Authors:** Hideto To, Mari Tomonari, Makoto Myojin, Fumiyasu Okazaki

**Affiliations:** 1 Department of Medical Pharmaceutics, Graduate School of Medicine and Pharmaceutical Sciences, University of Toyama, Toyama 930-0194, Japan; m.tomonari193@yakuto.net (M.T.); mako.yakugaku910@gmail.com (M.M.); 2 Toyama Community Medicine & Laboratory, Toyama 939-0287, Japan; 3 Department of Drug Analysis, Daiichi University of Pharmacy, Fukuoka 815-8511, Japan; f-okazaki@daiichi-cps.ac.jp

**Keywords:** irinotecan hydrochloride, SN-38, SN-38 glucuronide, delayed diarrhea, pharmacokinetics

## Abstract

**Background:** Irinotecan hydrochloride (CPT-11) is an important anticancer drug used in a wide range of regimens to treat colorectal and gastric cancers, and one of its severe side effects is delayed diarrhea. Therefore, based on known circadian variations in intestinal function and drug metabolism, we investigated whether CPT-11-induced delayed diarrhea may be attenuated by the time of dosing. **Methods:** When CPT-11 was administered to rats at 9:00 or 21:00, CPT-11-induced delayed diarrhea was assessed, and concentrations of CPT-11, its active metabolite SN-38, and SN-38 glucuronide (SN-38GL) in blood, intestinal tissues, and intestinal contents were measured. **Results:** The severity of diarrhea was significantly less in the 21:00 dosing group compared with the 9:00 dosing group. Blood SN-38 concentrations 8 h after the administration of CPT-11 were significantly higher in the 9:00 dosing group than in the 21:00 dosing group. SN-38, which exerts potent cytotoxic effects, circulates enterohepatically. When SN-38 is absorbed from the intestinal mucosa, intestinal tissues may be injured, resulting in delayed diarrhea. CPT-11 and SN-38 concentrations in intestinal tissues and contents 8 h after the administration of CPT-11 were significantly higher in the 9:00 dosing group than in the 21:00 dosing group at all measurement points. This was consistent with more severe CPT-11-induced delayed diarrhea in the 9:00 dosing group. **Conclusions**: Chronotherapy with CPT-11 may reduce CPT-11-induced delayed diarrhea. These differences in SN-38 concentrations in the intestinal tract at different dosing times may contribute to the time-dependent reduction in CPT-11-induced delayed diarrhea.

## 1. Introduction

Irinotecan hydrochloride (CPT-11) is an anticancer drug that is widely used to treat colon, stomach, and lung cancers [1,2]. It exerts its cell-killing effects by inhibiting topoisomerase I, which is responsible for cleaving one of the two strands of DNA and relaxing the DNA superhelix that occurs primarily during replication and transcription [3,4]. CPT-11 is metabolized by carboxylesterase in the liver and blood and is hydrolyzed to the active metabolite SN-38, the cytotoxicity of which is approximately 1000-fold higher than CPT-11 [5]. SN-38 is glucuronidated by UDP-glucuronosyltransferase 1A1 (UGT1A1) in the liver and converted to SN-38 glucuronide (SN-38GL), which is highly soluble in water, less cytotoxic, and excreted from the body [6]. Therefore, when considering the efficacy and safety of CPT-11, it is necessary to understand its pharmacokinetics, including these metabolites.

The dose-limiting factors of CPT-11 include leukopenia and delayed diarrhea [7,8]. In clinical practice, the severity of myelosuppression has been reduced by using granulocyte colony-stimulating factor (G-CSF) preparations and other agents [9]. On the other hand, adequate treatments for delayed diarrhea have yet to be established. SN-38GL, as well as CPT-11 and SN-38 excreted into the intestinal tract, play important roles in the pathogenesis of CPT-11-induced delayed diarrhea. SN-38GL is deconjugated to SN-38 by β-glucuronidase (β-GL) from enteric bacteria. Increases in SN-38 contribute to intestinal mucosal cell injury, resulting in delayed diarrhea [10]. Therefore, the prophylactic administration of oral alkalinizing agents [11], Kampo medicine (Hangeshashin-to) [12], and antibiotics [13] to inhibit SN-38GL deconjugation in the intestinal tract have been attempted. These trials have shown some benefits but have not yet resulted in prophylaxis suitable for treatment regimens due to the various challenges posed by these medications.

The circadian rhythm is a periodic variation with a cycle of approximately one day that exists in almost all living organisms, including humans, and is present in the activity rhythms of sleep and wakefulness, blood pressure, blood coagulation capacity, and the secretion of hormones, such as cortisol and melatonin [14,15]. These rhythms are also observed in diseases, with attacks in bronchial asthma and stiffness in rheumatoid arthritis generally occurring during specific time periods [16,17]. Chronotherapy is a treatment method in which drugs are administered at the optimum time, considering circadian rhythms, thereby increasing therapeutic efficacy and reducing side effects. We have researched chronopharmacology in animals and humans with various diseases, such as cancer and rheumatoid arthritis, and demonstrated that treatment efficacy and safety can be improved by optimizing the timing of drug administration during the day [18,19,20,21]. A previous study on CPT-11 chronopharmacology in mice reported that myelosuppression was markedly reduced when the optimal dosing time was selected [22]. However, whether dosing time affects delayed diarrhea caused by the administration of CPT-11 has yet to be clarified.

β-GL activity may be an important factor contributing to CPT-11-induced delayed diarrhea. A previous study reported that β-GL activity in the rat liver exhibited a distinct circadian rhythm with high levels during the dark phase and low levels during the light phase [23]. However, the diurnal rhythm of β-GL activity in intestinal contents remains unknown. Therefore, to clarify whether dosing time affects CPT-11-induced delayed diarrhea, we first investigated the circadian rhythm of β-GL activity in intestinal contents in rats. Based on the information obtained, we also set the dosing time and examined the effects of different dosing times on the severity of delayed diarrhea. In addition, drug concentrations in blood and intestinal tissues and contents were measured to elucidate the underlying mechanisms. To estimate the mechanisms underlying differences in drug concentrations within the intestinal tracts and contents, we measured mRNA levels of P-glycoprotein (*P-gp*) and multi-drug resistance protein 2 (*Mrp2*), which are drug transporters, in the liver and intestinal tracts (ileum, cecum and colon). Moreover, clock genes (*Bmal1*, *Clock*, *Cry1* and *Per2*) mRNA expression levels were measured in liver and intestinal tissues. Although previous studies examined chronomodulation of CPT-11 toxicity, the relationship between dosing time, intestinal exposure to CPT-11/SN-38, and delayed diarrhea has not been sufficiently investigated.

## 2. Materials and Methods

### 2.1. Reagents

CPT-11, SN-38, SN-38GL, and camptothecin (CPT) were obtained from Yakult Honsha Co., Ltd. (Tokyo, Japan), and CPT-11 was purchased from Selleck Biotechnology (Houston, TX, USA). p-Nitrophenyl-β-D-glucuronide was obtained from Sigma-Aldrich (St. Louis, MO, USA). β-GL was supplied by FUJIFILM Wako Pure Chemical (Osaka, Japan).

### 2.2. Animals

Seven-week-old male Wistar rats were purchased from SLC Japan (Hamamatsu, Japan). Rats were provided food and water ad libitum with a 12 h light–dark cycle (light period: 7:00–19:00) for 2 weeks before being used in experiments. The rats were randomly assigned to each experimental group. The dark-period experiment was conducted under red light. Animal experimental protocols were approved by the Animal Care and Use Committee at the University of Toyama and conducted in accordance with their Institutional Animal Experiment Handling Rules (A2011PHA-11, date of approval: 31 March 2011; A2014PHA-15, date of approval: 9 June 2014; A2023PHA-18, date of approval: 31 January 2024).

### 2.3. Measurement of β-GL Activity

Intestinal contents (ileum, cecum, and colon) were collected at 9:00, 13:00, 17:00, 21:00, 1:00, and 5:00 (*n* = 5). After collection, the samples were promptly stored at −80 °C until analyzed. Ultrasonic disruption was performed using 30 mL of phosphate-buffered solution for every 1 g of intestinal contents, with a cooling schedule of (20 s disruption + 20 s pause) × 12 times. The resulting suspension was then centrifuged at 4000× *g* at 4 °C for 10 min, and the supernatant was collected. The supernatant was diluted with phosphate buffer to make a sample solution. One hundred microliters of the sample solution were added to a 96-well plate and preincubated at 37 °C for 10 min. Fifty microliters of the substrate solution (p-nitrophenyl-β-D-glucuronide) was then added, and absorbance was measured at 405 nm at 37 °C for 15 min. [24]. The β-GL activity of each sample was calculated using the β-GL activity of the β-GL solution (FUJIFILM Wako Pure Chemical; from *Escherichia coli* (Lot: LTR 4400)) as an indicator. After confirming the linearity of the reaction, the increase in absorbance was compared with a standard curve prepared with *p*-nitrophenol. Enzyme activities were calculated in International Units (IU), where 1 IU is the amount of enzyme that hydrolyzes 1 μmol of substrate/min at 37 °C.

### 2.4. Experimental Design

In the CPT-11-induced diarrhea rat model, CPT-11 was administered intravenously by the tail vein once a day for 4 days, as reported in previous studies [13,25]. CPT-11 was used after adjusting the dose to 20 mg/mL with boiled water. Rats were administered CPT-11 (60 mg/kg) intravenously by the tail vein at 9:00 or 21:00 for four consecutive days to evaluate CPT-11-induced delayed diarrhea at different dosing times [13] (*n* = 12) and after a single administration to measure pharmacokinetics (*n* = 5–6).

### 2.5. Evaluation of Body Weight, Food Intake, and Delayed Diarrhea

Body weight and food intake were measured daily at 9:00 or 21:00 (*n* = 12). Changes in body weight were calculated as the percent weight change in each rat from the initial value (day 0).

Two types of CPT-11-induced diarrhea were considered per the definitions of early and delayed diarrhea reported by Takasuna et al. [13]. Early diarrhea was defined as occurring up to 3 h after administration, and delayed diarrhea as occurring between 6 and 24 h after administration. During the observation period, diarrhea was observed once a day and evaluated as delayed diarrhea. The diarrhea score was classified into four levels: 0 (normal stools), 1 (slightly moist and soft stools), 2 (moist and unformed feces, moderate anal discharge), and 3 (watery stools, severe anal discharge).

### 2.6. Sampling of Blood, Intestinal Tissues (Ileum, Cecum, and Colon) and Intestinal Contents (Ileum and Cecum)

Blood (200 μL) was collected from the tail vein at 0.083, 1, 2, 4, 6, 8, 12, 15, 19, and 24 h after CPT-11 administration at 9:00 or 21:00 (*n* = 5), and plasma was obtained. Intestinal tissues and contents of the ileum, cecum and colon were collected at 8 or 15 h after CPT-11 dosing (*n* = 6). Tissue and contents were collected from the ileum and colon, specifically a 10 cm segment of the ileum excluding the 10 cm section starting from the cecum, and a 10 cm segment of the colon excluding the 5 cm section starting from the cecum. These samples were stored at −20 °C until analysis.

### 2.7. Measurement of CPT-11, SN-38, and SN-38GL Concentrations

Plasma (20 μL) was spiked with 380 μL of an internal standard solution (CPT in DMSO, 4 ng/mL: 50% methanol in acetonitrile (20: 360)) and vortexed. All solutions were centrifuged at 14,800× *g* at 15 °C for 15 min. The supernatant (300 μL) was transferred to another tube and evaporated to dryness using a SpeedVac (Thermo Fisher Scientific, Waltham, MA, USA; temperature: 60 °C; heat time: 1.0 h; run time: 1.0 h; vacuum pressure: 5.1). The residue was reconstituted with 80 μL of 50% methanol in water (1:1).

Frozen intestinal tissues were thawed, chopped, and weighed at 4 °C. A total of 225 mg of chopped tissue was homogenized in 3 mL of ice-cold homogenizing solution containing 10 mM potassium phosphate (pH 7.4), 250 mM sucrose, and 1 mM EDTA using a polytron tissue homogenizer.

Homogenized samples (60 μL) were spiked with 1140 μL of an internal standard solution (CPT in DMSO, 1.33 ng/mL: 50% methanol in acetonitrile (20: 360)) and vortexed. All solutions were centrifuged at 14,800× *g* at 15 °C for 15 min. The supernatant (900 μL) was transferred to another tube and evaporated to dryness using a SpeedVac (temperature: 60 °C; heat time: 2.0 h; run time: 2.0 h; vacuum pressure: 5.1). The residue was reconstituted with 80 μL of 50% methanol in water (1:1).

Frozen intestinal contents were thawed and weighed at 4 °C. Thirty milligrams of each intestinal content was homogenized in 3 mL of ice-cold homogenizing solution using a polytron tissue homogenizer. Homogenized samples (20 μL) were spiked with 380 μL of an internal standard solution (CPT in DMSO, 4 ng/mL: 50% methanol in acetonitrile (20: 360)) and vortexed. All solutions were centrifuged at 14,800× *g* at 15 °C for 15 min. The supernatant (300 μL) was transferred to another tube and evaporated to dryness using a SpeedVac (temperature: 60 °C; heat time: 1.0 h; run time: 1.0 h; vacuum pressure: 5.1). The residue was reconstituted with 80 μL of 50% methanol in water (1:1).

### 2.8. UPLC

The chromatographic separation of CPT-11, SN-38, and SN-38GL was performed using the Vanquish UHPLC System (Thermo Fisher Scientific). Analyses were conducted using CORTECS UPLC C18, 2.1 × 50 mm (Waters, Milford, MA, USA). Mobile phase A (0.1% formic acid in water [*v*/*v*]) and mobile phase B (100% acetonitrile) were operated with gradient elution at a flow rate of 0.4 mL/min as follows: 10% B → 25% B (0–0.5 min), 25% B → 40% B (0.5–1 min), 40% B (1–2.5 min), and 40% B → 10% B (2.5–4.5 min). Column and sample temperatures were 40 °C and 20 °C. The injection volume was 10 μL.

### 2.9. Mass Spectrometry

Measurements of CPT-11, SN-38, and SN-38GL were based on Basu et al. [26] and LC–MS/MS analysis was performed using a Q Exactive Plus Orbitrap mass spectrometer (Thermo Fisher Scientific). Mass spectrometry conditions were a heating-type electrospray ionization source; spray voltage, (+) 3500 V; capillary temperature, 263 °C; sheath gas flow rate, 50 arb units; auxiliary gas flow rate, 13 arb units; sweep gas flow rate, 3 arb units; and auxiliary gas heater temperature, 425 °C. Product ions were selected based on their significance in the MS/MS spectra. Data were processed using Analyst Software. Analyses were performed in positive ion electrospray mode using parallel reaction monitoring (PRM) acquisition mode. The precursor ions (*m*/*z*) selected for each compound were as follows: CPT-11, *m*/*z* 587; SN-38, *m*/*z* 393; SN-38GL, *m*/*z* 569; and CPT, *m*/*z* 349. Product ions were obtained via high-energy collision-induced dissociation (HCD, normalized collision energy 25 or 35) and monitored at high resolution using an Orbitrap mass analyzer as follows: CPT-11, *m*/*z* 124; SN-38, *m*/*z* 349; SN-38GL, *m*/*z* 393; and CPT, *m*/*z* 305.

### 2.10. Measurement of mRNA Expression Levels of Efflux Transports (P-gp and Mrp2) and Clock Genes (Bmal1, Clock, Cry1 and Per2) in Liver and Intestinal Tissues

Liver and intestinal tissues (ileum, cecum, and colon) were collected at 9:00, 13:00, 17:00, 21:00, 1:00, and 5:00 (*n* = 6). After collection, the samples were promptly stored at −80 °C until analyzed. Total mRNA was extracted using RNAiso Plus (TaKaRa Bio Inc., Kusatsu, Japan), and cDNA was synthesized using PrimeScript FAST RT Reagent Kit with gDNA Eraser (TaKaRa Bio Inc.). Real-time PCR analyses were performed using GoTaq qPCR Master Mix (Promega, Madison, WI, USA) and LightCycler 96 (Roche Diagnostics, Mannheim, Germany).

The gene expression values represent the normalized expression levels of each sample (raw gene expression level/18S rRNA gene expression level) relative to the average gene expression level across all samples.

### 2.11. Statistical Analysis

Food intake, β-GL activity, and drug concentrations are shown as mean ± standard deviation (S.D.), and other values are expressed as mean ± standard error (S.E.). Groups were compared using one-way analysis of variance (ANOVA), and differences between groups were examined by Scheffe’s test. Differences between groups were analyzed using Student’s *t*-test. Circadian rhythmicity was analyzed using the Cosinor method by fitting the data to a 24 h cosine wave. The key parameters, including acrophase and amplitude along with the *p*-values for rhythmicity using Time Series Analysis Cosinor 8.0 (France). We considered 24 h rhythmicity to be significant when both Cosinor analysis and the one-way ANOVA were significant. Diarrhea scores among groups were compared using the Kruskal–Wallis test, and post hoc testing was conducted using the Mann–Whitney U test with Bonferroni correction for non-parametric data.

A probability level of less than 0.05 was considered significant. All statistical analyses, except for the Cosinor method, were performed using IBM SPSS Statistics software (version [28.0.1.1], IBM Corp., Armonk, NY, USA).

## 3. Results

### 3.1. Circadian Rhythms of β-GL Activity in Intestinal Contents

**Figure 1:**
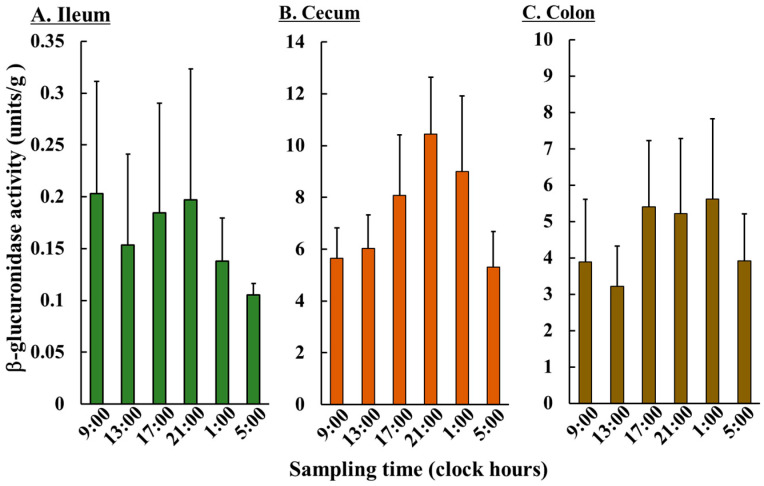
Circadian rhythms of β-GL activity in intestinal contents: (**A**) ileum, (**B**) cecum, and (**C**) colon. Each value is the mean ± S.D. of five rats. In the content of the cecum, β-GL activity showed a significant 24 h rhythm (ANOVA and Cosinor: *p* < 0.01, respectively). The bars highlighted in green, orange, and brown represent the ileum, cecum, and colon, respectively. Data on the key parameters (amplitude, and acrophase) for β-GL activity were included in Supplementary Data S1.

Figure 1 shows β-GL activities in the ileum, cecum, and colon. β-GL activities in the contents of the ileum and colon did not show significant diurnal variations. In the cecum, a significant circadian rhythm was observed for β-GL activity, with a high value at 21:00, and a low value at 5:00 (ANOVA and Cosinor: *p* < 0.01, respectively). Comparisons among the three intestinal tissues revealed that β-GL activity was highest in the cecum, followed by the colon; the ileum exhibited approximately one-fiftieth the activity of the other two sites.

### 3.2. Effects of CPT-11 Dosing Time on Food Intake and Body Weight

**Figure 2:**
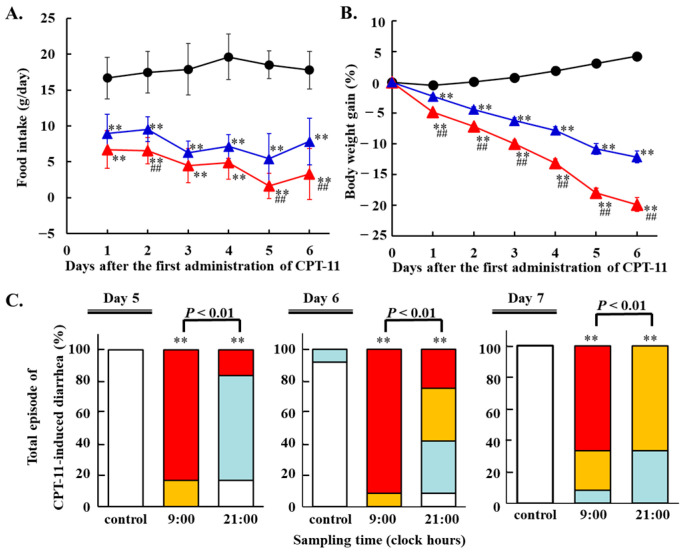
Change in food intake and body weight, and incidence of delayed diarrhea in rats after intravenous administration of CPT-11 at 9:00 or 21:00 at a consecutive daily dose of 60 mg/kg (*n* = 12). (**A**,**B**) Each value in food intake and body weight is the mean ± S.D. The black closed circle is the control group, the red triangle is the 9:00-treated group, and the blue triangle is the 21:00-treated group. ** *p* < 0.01 for control group vs. 9:00-treated or 21:00-treated group; ## *p* < 0.01 for 9:00-treated group vs. 21:00-treated group. (**C**) Diarrhea score was classified into four levels: 0 (normal stools; white column), 1 (slightly moist and soft stools; light blue column), 2 (moist and unformed feces, moderate anal discharge; orange column), and 3 (watery stools, severe anal discharge; red column). Food intake and body weight were markedly lower in the 9:00-treated group than in the control and 21:00-treated groups. During all observation periods, the 9:00 dosing group experienced severe delayed diarrhea compared with the 21:00 dosing group (*p* < 0.01, respectively).

Food intakes in the CPT-11-treated groups were less than half that of the control group from the day after treatment began (*p* < 0.01, respectively, Figure 2A). The 9:00 dosing group consumed a maximum of only 8.9% of the control group’s food intake on day 5. Food intake was significantly lower on days 2, 5, and 6 in the 9:00 dosing group compared with the 21:00 dosing group (*p* < 0.01, respectively).

After four consecutive days of intravenous CPT-11 dosing at 9:00 or 21:00, body weight was significantly lower in both the 9:00 and 21:00 dosing groups than in the control group (*p* < 0.01, respectively, Figure 2B). Furthermore, more severe weight loss was observed in the 9:00 dosing group than in the 21:00 dosing group (*p* < 0.01, respectively).

### 3.3. Effects of CPT-11 Dosing Time on Diarrhea Scores

Delayed diarrhea, defined as occurring from 6 h after the last administration, was evaluated on days 5, 6, and 7. Median diarrhea scores were 3, 3, and 3, respectively, in the 9:00 dosing group, and 1, 2, and 2, respectively, in the 21:00 dosing group. Both dosing groups showed significantly higher diarrhea scores than the control group at all observed points (*p* < 0.01, respectively, Figure 2C). The severity of delayed diarrhea was higher in the 9:00 dosing group than in the 21:00 dosing group (*p* < 0.01, respectively).

### 3.4. Effects of CPT-11 Dosing Time on Blood CPT-11, SN-38, and SN-38GL Concentrations

**Figure 3:**
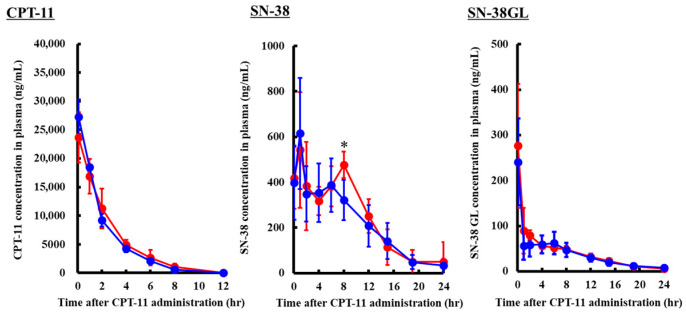
Effects of dosing time on blood CPT-11, SN-38, and SN-38GL concentrations after a single administration of CPT-11 at 9:00 (red circle) or 21:00 (blue circle) in rats. Each value is the mean ± S.D. of five rats. Eight hours after CPT-11 administration, blood SN-38 concentration was approximately 1.48 times higher in the 9:00 dosing group than in the 21:00 dosing group. (* *p* < 0.05 for 9:00-treated group vs. 21:00-treated group).

The lower limit of detection (LLOD) and upper limit of quantification (ULOQ) in plasma were 5 and 5000 ng/mL for CPT-11, 7.81 and 1000 ng/mL for SN-38, and 3.91 and 250 ng/mL for SN-38GL. Changes in blood CPT-11, SN-38, and SN-38GL concentrations over time after CPT-11 dosing at 9:00 or 21:00 are shown in Figure 3. It was not possible to measure CPT-11 concentrations from the 15th hour after CPT-11 dosing because they were below the lower limit of quantification. Blood CPT-11 and SN-38GL concentrations did not significantly differ at any time point between the 9:00 and 21:00 dosing groups. On the other hand, blood SN-38 concentrations 8 h after dosing were significantly higher in the 9:00 dosing group than in the 21:00 dosing group (*p* < 0.05).

### 3.5. Effects of CPT-11 Dosing Time on CPT-11, SN-38, and SN-38GL Concentrations in Ileum, Cecum, and Colon Tissues and Ileum and Cecum Contents

The LLOQ and ULOQ in tissues were 0.67 and 66.67 μg/g for CPT-11, 0.1 and 4.0 μg/g for SN-38, and 0.052 and 0.833 μg/g for SN-38GL. The LLOQ and ULOQ of the contents were 5 and 500 μg/g for CPT-11, 0.75 and 30 μg/g for SN-38, and 0.39 and 25 μg/g for SN-38GL. Table 1 and Table 2 show CPT-11, SN-38, and SN-38GL concentrations in ileum, cecum and colon tissues and ileum and cecum contents 8 and 15 h after dosing with CPT-11 at 9:00 or 21:00.

CPT-11, SN-38, and SN-38GL concentrations in ileum tissue 8 h after dosing were significantly higher (approximately 2-fold) in the 9:00 dosing group than in the 21:00 dosing group (*p* < 0.01, respectively, Table 1). CPT-11, SN-38, and SN-38GL concentrations 15 h after dosing were similar in the 9:00 and 21:00 dosing groups (Table 2).

SN-38GL concentrations in ileum contents 8 h after dosing did not significantly differ between groups (Table 1). On the other hand, CPT-11 and SN-38 concentrations were 2.97- and 2.04-fold higher, respectively, in the 9:00 dosing group than in the 21:00 dosing group. Fifteen hours after dosing, CPT-11, SN-38, and SN-38GL concentrations were similar in the 9:00 and 21:00 dosing groups (Table 2).

In cecum tissue, CPT-11 and SN-38 concentrations 8 h after dosing were approximately 2-fold higher in the 9:00 dosing group than in the 21:00 dosing group (*p* < 0.01, respectively, Table 1). SN-38GL concentrations did not significantly differ between the two groups. CPT-11 and SN-38 concentrations in cecum tissue 15 h after dosing were also slightly higher in the 9:00 dosing group than in the 21:00 dosing group (Table 2). No significant differences were observed in SN-38GL concentrations between the two groups; however, mean and median concentrations were 0.082 and 0.076 μg/g, respectively, in the 9:00 dosing group and 0.229 and 0.13 μg/g, respectively, in the 21:00 dosing group, with lower concentrations being observed in the former.

It was not possible to measure SN-38GL concentrations in cecum contents 8 and 15 h after dosing because they were below the lower limit of quantification (Table 1 and Table 2). CPT-11 and SN-38 concentrations in cecum contents 8 h after dosing were significantly higher in the 9:00 dosing group than in the 21:00 dosing group (*p* < 0.01, respectively), whereas those 15 h after dosing did not significantly differ between the groups (Table 1).

CPT-11 and SN-38 concentrations in colon tissue 8 and 15 h after dosing were both significantly higher in the 9:00 dosing group than the 21:00 dosing group (*p* < 0.01, respectively, Table 1 and Table 2). SN-38GL concentrations in colon tissue were 2.26-fold higher in the 21:00 dosing group than in the 9:00 dosing group 8 h after dosing (*p* < 0.05) and 1.52-fold higher in the 9:00 dosing group than in the 21:00 dosing group 15 h after dosing.

### 3.6. Circadian Rhythms of mRNA Expression Levels of Transports (P-gp and Mrp2) and Clock Genes (Bmal1, Clock, Cry1 and Per2) in Liver and Intestinal Tissues

**Figure 4:**
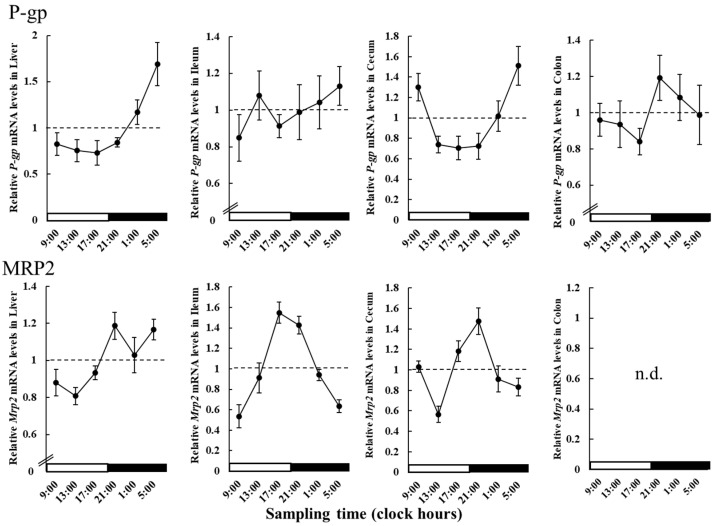
Circadian Rhythms of mRNA expression levels of transports (*P-gp* and *Mrp2*) in liver and intestinal tissues in rats. Each value is the mean ± S.E. of six rats. The dashed black line indicates the baseline value of 1. The white and black bars at the bottom of the graph represent the light and dark phases, respectively. The *P-gp* mRNA expression levels showed significant circadian rhythms with peak at 5:00 in the liver and cecum (*p* < 0.001, respectively). There were circadian rhythms in the *Mrp2* mRNA levels in the liver, ileum and cecum with high levels during the early dark phase and low levels during the light phase *p* < 0.01 and 0.001, respectively). Data on the key parameters (amplitude, and acrophase) for RNA expression levels of efflux transporters (*P-gp* and *MRP2*) were included in Supplementary Data S1.

**Figure 5:**
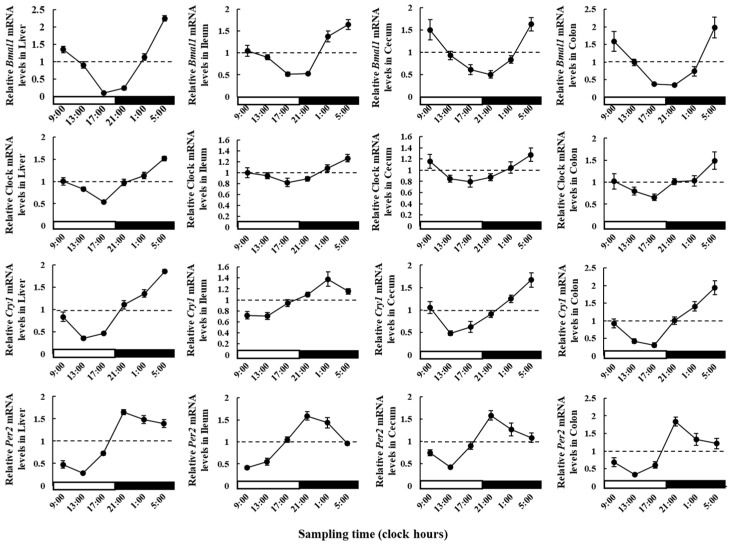
Circadian Rhythms of mRNA expression levels of clock genes (*Bmal1*, *Clock*, *Cry1* and *Per2*) in liver and intestinal tissues. Each value is the mean ± S.E. of six rats. The dashed black line indicates the baseline value of 1. The white and black bars at the bottom of the graph represent the light and dark phases, respectively. In the liver, ileum, cecum and colon, there were 24 h rhythms in *Bmal1*, *Clock*, *Cry1* and *Per2* mRNA expression (*p* < 0.05, 0.01 and 0.001, respectively). Data on the key parameters (amplitude, and acrophase) for RNA expression levels of efflux transporters (*Bmal1*, *Clock*, *Cry1*, *Per2*) were included in Supplementary Data S1.

Figure 4 and Figure 5 show 24 h rhythms of *P-gp*, *Mrp2*, *Bmal1*, *Clock*, *Cry1* and *Per2* mRNA levels in liver, ileum, cecum, and colon.

The mRNA expression levels of *P-gp* showed significant circadian rhythms with a peak at 5:00 and trough at 17:00 in the liver and cecum (ANOVA and Cosinor: *p* < 0.001, respectively; Figure 4). The *Mrp2* mRNA levels in liver, ileum and cecum exhibited distinct circadian rhythms with high levels during the early dark phase and low levels during the light phase (ANOVA and Cosinor: *p* < 0.01, respectively; Figure 4). In colon tissue, the mRNA expression levels of *Mrp2* were below the limit of quantification and therefore could not be determined. In all sampled organs (liver, ileum, cecum, and colon), the mRNA expression levels of all measured clock genes (*Bmal1*, *Clock*, *Cry1* and *Per2*) showed significant 24 h rhythms (ANOVA and Cosinor: *p* < 0.05, 0.01 and 0.001, respectively; Figure 5).

## 4. Discussion

In the present study, we measured β-GL activity and its circadian rhythm in ileum, cecum, and colon contents. Average β-GL activity (units/g) in one day was 0.16 in the ileum, 7.42 in the cecum, and 4.54 in the colon, with β-GL activity in the ileum being the lowest. Injury to the intestinal epithelial tissue of rats has been shown to be more severe in the cecum, followed by the colon and ileum [25]. This severity of intestinal injury corresponds to β-GL activity at each site in the present study, suggesting that β-GL activity may significantly contribute to the severity of CPT-11-induced delayed diarrhea. A distinct circadian rhythm in β-GL activity was observed in cecum contents, with low values at 5:00 and 9:00 and the highest value at 21:00. In colon contents, similar to cecum contents, a diurnal variation was observed, with low values during the light phase and high values during the dark phase; however, this variation was not significant. We hypothesized that timing CPT-11 administration according to the circadian rhythm may alleviate CPT-11-induced delayed diarrhea. Therefore, we examined the effects of CPT-11 on delayed diarrhea by setting the CPT-11 administration time to 21:00, when β-GL activity in the cecum peaks, and also at 9:00, 12 h later.

Delayed diarrhea was assessed based on the condition of stools excreted from the 6 to 24 h after CPT-11 administration, as previously described [13]. Preliminary investigations showed that the incidence of delayed diarrhea was approximately 60% on the first day of administration, increased over time, and was 100% after day 5. The median severity of diarrhea was 1 until day 4, 3 after day 5, and continued to increase thereafter. According to this model, we examined the effects of different CPT-11 dosing times on delayed diarrhea. From the first day of treatment, food intake was markedly lower in both dosing groups than in the control group, with reductions of up to approximately 90% noted. On the day after the last dose of CPT-11 (day 5), food intake in the 9:00 dosing group was approximately half that in the 21:00 dosing group, suggesting that side effects were more severe in the 9:00 dosing group. Body weight over time was also significantly lower in the 9:00 dosing group than in the 21:00 dosing group from the beginning of CPT-11 administration. Although delayed diarrhea occurred in both dosing groups, it was more severe in the 9:00 dosing group than in the 21:00 dosing group. Therefore, the severity of CPT-11-induced delayed diarrhea may be attenuated by selecting the optimal dosing time. Although the circadian rhythm of β-GL activity in the intestines was considered to contribute to diarrhea onset, delayed diarrhea was mild at 21:00 when β-GL activity was the highest. This suggests that β-GL activity alone is insufficient to explain the severity of delayed diarrhea. We hypothesized that the pharmacokinetics of CPT-11 may explain this apparent discrepancy between the diurnal variation in β-GL activity and the level of toxicity. Moreover, E4bp4 and Rev-erbα, both of which are circadian clock genes, have been shown to regulate the expression of carboxylesterase 2 (CES2), a key enzyme involved in CPT-11 metabolism. E4bp4 suppresses the expression of CES2 by inhibiting Rev-erbα, suggesting that the enzymatic conversion of CPT-11 to SN-38 is under circadian control [27]. These findings indicate that fluctuations in SN-38 levels due to the circadian regulation of CES2 affect the timing and severity of intestinal toxicity. Since SN-38 is the primary factor causing intestinal injury, the metabolism of CPT-11 to SN-38 and SN-38GL, the elimination of SN-38 and SN-38GL from the blood to the intestinal tract, and the deconjugation of SN-38GL to SN-38 were unlikely to have led to the development of toxicity immediately after CPT-11 dosing. Therefore, a lag likely exists between the onset of toxicity after drug administration, prompting examination of the effects of different dosing times of CPT-11 on blood CPT-11, SN-38, and SN-38GL concentrations.

Blood CPT-11 and SN-38GL concentrations did not significantly differ between the 9:00 and 21:00 dosing groups. On the other hand, blood SN-38 concentrations 8 h after CPT-11 dosing were approximately 1.5-fold higher in the 9:00 dosing group than in the 21:00 dosing group. These results indicate that the plasma concentration of SN-38, which mainly causes tissue injury in the intestinal tract, changed depending on the time of dosing. However, since the pharmacokinetics of CPT-11 and SN-38GL did not change with dosing time, the change in SN-38 pharmacokinetics at different dosing times did not appear to result from the metabolism of CPT-11 to SN-38 or SN-38 to SN-38GL in the liver. SN-38, the active metabolite of CPT-11, circulates enterohepatically [10]. Therefore, the development of CPT-11-induced delayed diarrhea may involve injury to intestinal tissues when SN-38, which is highly cytotoxic, is absorbed from the intestinal mucosa. We hypothesized that the increase in the blood SN-38 concentration that occurred from 4 to 15 h after CPT-11 dosing was due to the enterohepatic circulation of SN-38, and also that the intestinal concentration of SN-38 8 h after dosing was higher in the 9:00 dosing group than in the 21:00 dosing group. We focused on the 8th hour after dosing, when differences in plasma SN-38 concentrations occur, and the 15th hour after dosing, when these differences disappear, and examined the effects of different dosing times on CPT-11, SN-38, and SN-38GL concentrations in intestinal tissues and contents, the sites of injury by CPT-11 dosing. However, drug concentrations in the large intestine were not measured because some rats did not have contents in the colon tissue collection site and, thus, sufficient samples were not collected.

Eight hours after the administration of CPT-11, CPT-11 and SN-38 concentrations in intestinal tissues and their contents were approximately 2-fold higher in the 9:00 dosing group than in the 21:00 dosing group. Fifteen hours after the administration of CPT-11, CPT-11 and SN-38 concentrations in cecum and colon tissues remained approximately 2-fold higher in the 9:00 dosing group than in the 21:00 dosing group. These results indicate that the 9:00 dosing group was exposed to higher concentrations of CPT-11 and SN-38 in cecum and colon tissues, where damage is more pronounced at the onset of delayed diarrhea. The higher concentration of SN-38 in blood at 8 h may be due to the reabsorption of SN-38 in intestinal tissues and contents, particularly in the 9:00 dosing group. Furthermore, these differences in drug concentrations at the dosing times examined corresponded to varying severities of delayed diarrhea among intestinal tracts as mentioned previously [25]. Therefore, intestinal pharmacokinetics may play an important role in the observed differences in the severity of delayed diarrhea at the CPT-11 dosing times examined.

CPT-11, SN-38, and SN-38GL are excreted in bile from the liver into the intestinal tract mainly via P-gp and MRP2, and are then excreted out of the body in feces [10]. Some may also be reabsorbed from the intestinal tract via enterohepatic circulation. Circadian changes in the pharmacokinetics of these drugs in the intestinal tract are thought to involve efflux transporters during drug excretion into the intestinal tracts, intestinal peristalsis to excrete feces and other substances from the body, and β-GL activity derived from intestinal bacteria. Previous studies reported diurnal rhythms for P-gp and MRP2 proteins in the mouse liver, with low levels during the light phase and high levels during the dark phase [28,29]. In this study, the mRNA levels of *P-gp* and *Mrp2* in the liver exhibited a circadian rhythm with a peak during the dark phase consistent with previous reports. The CPT-11 and SN-38 levels in the intestinal tissues and contents at 8 h after the drug dosing were higher in the 9:00-treated group than in the 21:00-treated group, despite previous reports indicating lower hepatic transporter levels during the light phase. These findings do not show a clear concordance between the circadian rhythms of hepatic transporters and the drug concentrations in intestinal tissues and contents in our experimental setting, suggesting that hepatic transporter rhythmicity alone is insufficient to fully explain the observed pharmacokinetic differences.

Transporters are involved not only in the biliary excretion of drugs from the liver into the intestinal tract but also in drug efflux processes in the intestine. The P-gp protein in the mouse ileum has a diurnal rhythm with low levels in the light phase and high levels in the dark phase [29]. In this study, as in previous reports, a distinct circadian rhythm in the mRNA levels of *P-gp* and *Mrp2* was observed in the intestine. A previous study had reported that circadian oscillations of *Mrp2* mRNA are accompanied by corresponding rhythms in MRP2 protein expression and transporter-mediated drug disposition in the intestine. In particular, the circadian rhythm of intestinal MRP2 expression was associated with time-dependent differences in methotrexate accumulation in intestinal tissues and systemic drug exposure [30]. Therefore, although only mRNA expression was evaluated in the present study, rhythmic changes in transporter function may also have contributed to the observed dosing-time-dependent differences in intestinal CPT-11 and SN-38 exposure. This suggests that, in the 21:00 dosing group, CPT-11 was administered during the dark phase when transporter expression is high, resulting in greater drug efflux from the intestinal tissue into the intestinal lumen; consequently, drug concentrations in the intestinal tissues were lower than those in the 9:00 dosing group. The drug concentration in tissue is a key factor determining the extent of cellular damage to the intestinal tissues caused by CPT-11 and SN-38. Previous reports have shown that damage to rat intestinal tissue following CPT-11 administration is most severe in the cecum, followed by the colon and then the ileum [25], and it is mentioned that SN-38 concentration in particular contributes to this toxicity [10]. This is consistent with the SN-38 concentrations observed in each tissue 8 h after CPT-11 administration in this study, with the highest concentration found in the cecum across all dosing time groups. Furthermore, when examining SN-38 concentrations in various tissues at different dosing-time points after dosing, the levels in the 9:00-treated group, which exhibited severe delayed diarrhea, were approximately twice as high as those in the 21:00-treated dosing group in all tissues. Based on the above, these results suggest that the differences in the severity of delayed diarrhea observed depending on the time of administration are influenced by differences in SN-38 concentrations in intestinal tissue. Furthermore, the circadian rhythm of transporters in intestinal tissue is considered to be one contributing factor. Although β-GL activity peaked at 21:00, delayed diarrhea was attenuated at this dosing time, suggesting that β-GL activity alone does not determine toxicity severity and that intestinal exposure to CPT-11 and SN-38 may play a more dominant role. However, transporter protein expression and functional activity were not evaluated in the present study. Therefore, causal relationships between transporter rhythmicity and intestinal drug exposure remain to be clarified. Fecal excretion of CPT-11 and its metabolites was not evaluated in the present study; therefore, the contribution of luminal elimination and enterohepatic circulation to the observed time-dependent differences requires further investigation. The present study compared only two dosing times and therefore does not fully characterize the entire circadian susceptibility profile of CPT-11-induced delayed diarrhea. Because pharmacokinetic analyses were conducted after a single administration of CPT-11, it remains unclear whether similar pharmacokinetic differences persist under repeated dosing conditions used for diarrhea evaluation.

Circadian regulation of intestinal transporters may also be linked to molecular clock mechanisms. Previous studies have reported that the core clock gene Bmal1 regulates the expression of the intestinal efflux transporter MRP2 and influences drug disposition in mice [30]. In the present study, circadian rhythmicity of clock gene expression was also observed in intestinal tissues, suggesting that local molecular clock mechanisms are present in the intestine. These findings suggest that clock gene oscillations may contribute to the rhythmic expression of transporters involved in intestinal drug handling. However, functional links were not directly examined in this study.

Directly observable phenomena such as sleep–wake cycles and feeding behavior, as well as indirectly measured parameters like blood leukocyte counts and metabolic enzymes, exhibit circadian rhythms. Because circadian phase relationships differ between nocturnal rodents and diurnal humans, the optimal dosing time in clinical settings cannot be directly inferred from the present findings. In general, the active and resting phases of rodents are approximately 12 h out of phase with those of humans; however, the optimal administration timing does not always shift uniformly across all drugs and disease conditions. Indeed, previous chronopharmacological studies of certain medications, including treatments for rheumatoid arthritis, have demonstrated similar optimal dosing times between rodents and humans [20,21,31]. Therefore, clinical chronopharmacological studies are required to determine the most appropriate dosing schedule for CPT-11 in patients. In addition, the feasibility of chronomodulated CPT-11 administration should be evaluated in the context of current combination chemotherapy regimens and clinical practice. In addition, CPT-11 and SN-38 exist in equilibrium between their lactone (closed-ring) and carboxylate (open-ring) forms, which may influence their pharmacological activity and toxicity. Although we did not examine this interconversion in the present study, its stability warrants consideration in future chronopharmacological investigations.

## 5. Conclusions

The present study showed that the severity of delayed diarrhea varied with the dosing time of CPT-11. These findings suggest that dosing-time-dependent changes in the intestinal pharmacokinetics of CPT-11 and SN-38 play a key role in modulating the severity of delayed diarrhea. Furthermore, the circadian rhythm of transporters in the intestine may be one factor contributing to these changes. The application of chronotherapy for CPT-11-induced delayed diarrhea, for which no evidence-based prophylaxis has been established in clinical practice, is expected to contribute to improvements in the quality of life of patients and the smooth implementation of cancer treatment. The chronotherapy of CPT-11 could improve patient quality of life and enable more consistent cancer treatment.

## Figures and Tables

**Table 1:** CPT-11, SN-38, and SN-38GL concentrations in intestinal contents and tissues 8 h after CPT-11 dosing.

Dosing Time	Ileum Contents			Cecum Contents					
	CPT-11 (μg/g)	SN-38 (μg/g)	SN-38GL (μg/g)	CPT-11 (μg/g)	SN-38 (μg/g)	SN-38GL (μg/g)			
9:00	229.2 ± 94.8	5.03 ± 1.60	16.1 ± 5.0	390.2 ± 69.6	16.91 ± 2.19	n.d.			
21:00	80.0 ± 19.6	2.46 ± 1.72	11.1 ± 7.8	191.5 ± 96.5	8.79 ± 1.43	n.d.			
*p* values	*p* < 0.05	*p* < 0.05		*p* < 0.01	*p* < 0.01				
Dosing time	ileum tissue			cecum tissue			colon tissue		
	CPT-11 (μg/g)	SN-38 (μg/g)	SN-38GL (μg/g)	CPT-11 (μg/g)	SN-38 (μg/g)	SN-38GL (μg/g)	CPT-11 (μg/g)	SN-38 (μg/g)	SN-38GL (μg/g)
9:00	94.2 ± 20.8	1.21 ± 0.23	0.99 ± 0.37	52.2 ± 15.1	2.70 ± 0.88	0.16 ± 0.05	60.5 ± 5.6	1.63 ± 0.66	0.27 ± 0.20
21:00	45.6 ± 19.0	0.69 ± 0.15	0.27 ± 0.15	22.1 ± 8.5	1.21 ± 0.51	0.20 ± 0.06	25.7 ± 5.9	0.49 ± 0.17	0.61 ± 0.25
*p* values	*p* < 0.01	*p* < 0.01	*p* < 0.01	*p* < 0.01	*p* < 0.01		*p* < 0.01	*p* < 0.01	*p* < 0.05

Each value is the mean ± S.D. of 6 rats. Not detected: n.d.

**Table 2:** CPT-11, SN-38, and SN-38GL concentrations in intestinal contents and tissues 15 h after CPT-11 dosing.

Dosing Time	Ileum Contents			Cecum Contents					
	CPT-11 (μg/g)	SN-38 (μg/g)	SN-38GL (μg/g)	CPT-11 (μg/g)	SN-38 (μg/g)	SN-38GL (μg/g)			
9:00	72.2 ± 80.8	1.84 ± 1.22	9.20 ± 3.62	91.1 ± 49.0	7.91 ± 2.83	n.d.			
21:00	71.1 ± 34.3	1.79 ± 0.74	7.23 ± 5.88	88.7 ± 51.6	7.80 ± 2.63	n.d.			
*p* values									
Dosing time	ileum tissue			cecum tissue			colon tissue		
	CPT-11 (μg/g)	SN-38 (μg/g)	SN-38GL (μg/g)	CPT-11 (μg/g)	SN-38 (μg/g)	SN-38GL (μg/g)	CPT-11 (μg/g)	SN-38 (μg/g)	SN-38GL (μg/g)
9:00	16.8 ± 3.9	0.42 ± 0.09	0.28 ± 0.14	11.7 ± 3.5	1.16 ± 0.38	0.08 ± 0.03	15.19 ± 4.81	0.91 ± 0.24	0.44 ± 0.25
21:00	14.0 ± 4.4	0.51 ± 0.28	0.21 ± 0.12	7.7 ± 2.8	0.80 ± 0.30	0.23 ± 0.23	6.43 ± 3.26	0.38 ± 0.28	0.29 ± 0.17
*p* values				*p* = 0.054			*p* < 0.01	*p* < 0.01	

Each value is the mean ± S.D. of 6 rats. Not detected: n.d.

## Data Availability

Data presented in this study is contained within the article and Supplementary Materials. Further inquiries can be directed to the corresponding author.

## Supplementary Materials

The following supporting information can be downloaded at: https://www.mdpi.com/article/10.3390/pharmaceutics18060645/s1. Supplementary Data S1: Key parameters of Cosinor analysis.

## Abbreviations

CPT-11	Irinotecan hydrochloride
SN-38GL	SN-38 glucuronide
UGT1A1	UDP-glucuronosyltransferase 1A1
G-CSF	Granulocyte colony-stimulating factor
β-GL	β-glucuronidase
CPT	Camptothecin
CES2	Carboxylesterase 2
*P-gp*	P-glycoprotein
*MRP2*	Multidrug resistance-associated protein 2
