# Supplementary material for: Irinotecan Hydrochloride Administration Considering Dosing-Time Attenuates Delayed Diarrhea in Rats
Source: Pharmaceutics. 2026 May 24;18(6):645. doi: 10.3390/pharmaceutics18060645 (PMC13305351; doi:10.3390/pharmaceutics18060645)
Supplement: Supplementary file 1 [file pharmaceutics-18-00645-s001.zip › pharmaceutics-4258134-supplementary.pdf]

Supplementary Data S1 Key parameters of Cosinor analysis

	tissues	P value	amplitude	acrophase
β-glucuronidase activity	Caecum	$P < 0.01$	2.56 (±0.51)	-5.52 (±0.20)
P-glycoprotein (P-gp) mRNA	Liver	$P < 0.001$	0.41 (±0.09)	-1.00 (±0.22)
	Caecum	$P < 0.001$	0.42 (±0.08)	-1.52 (±0.19)
multi-drug resistance protein 2 (MRP2) mRNA	Liver	$P < 0.01$	0.17 (±0.04)	-0.14 (±0.25)
	Ileum	$P < 0.001$	0.52 (±0.06)	-4.98 (±0.11)
	Caecum	$P < 0.01$	0.26 (±0.08)	-5.49 (±0.29)
Bmal1 mRNA	Liver	$P < 0.001$	0.98 (±0.07)	-1.58 (±0.07)
	Ileum	$P < 0.001$	0.54 (±0.06)	-1.33 (±0.12)
	Caecum	$P < 0.001$	0.58 (±0.08)	-1.88 (±0.13)
	Colon	$P < 0.001$	0.82 (±0.11)	-1.86 (±0.13)
Clock mRNA	Liver	$P < 0.001$	0.39 (±0.04)	-1.10 (±0.11)
	Ileum	$P < 0.001$	0.19 (±0.04)	-1.27 (±0.21)
	Caecum	$P < 0.001$	0.24 (±0.06)	-1.41 (±0.23)
	Colon	$P < 0.001$	0.32 (±0.08)	-1.11 (±0.24)
Cry1 mRNA	Liver	$P < 0.001$	0.69 (±0.06)	-0.75 (±0.08)
	Ileum	$P < 0.001$	0.33 (±0.05)	-0.11 (±0.05)
	Caecum	$P < 0.001$	0.53 (±0.07)	-0.96 (±0.13)
	Colon	$P < 0.001$	0.65 (±0.09)	-0.02 (±0.14)
Per2 mRNA	Liver	$P < 0.001$	0.72 (±0.05)	-0.06 (±0.07)
	Ileum	$P < 0.001$	0.60 (±0.04)	-5.89 (±0.07)
	Caecum	$P < 0.001$	0.49(±0.06)	-6.15 (±0.13)
	Colon	$P < 0.001$	0.65 (±0.09)	-0.02 (±0.14)

mean (± standard error).
